# Anophthalmia, Global Developmental Delay, and Severe Dysphagia in a Young Girl With 14q22q23 Microdeletion Syndrome

**DOI:** 10.7759/cureus.16395

**Published:** 2021-07-14

**Authors:** Jeslin Kera, Pankaj Watal, Syed A Ali

**Affiliations:** 1 Medicine, University of Central Florida College of Medicine, Orlando, USA; 2 Radiology, Nemours Children's Hospital, Orlando, USA; 3 Inpatient Pediatrics, Nemours Children's Hospital, Orlando, USA

**Keywords:** 14q22q23 microdeletion syndrome, frias syndrome, anophthalmia, global developmental delay, chromosome 14, facial dysmorphism, bmp4, otx2, six1, six6

## Abstract

14q22q23 microdeletion syndrome, also called Frias syndrome, is an extremely rare partial deletion of the long arm of chromosome 14 characterized by the anomalies of the pituitary gland, eyes, and hand/foot. Intellectual disability and facial dysmorphism are other common manifestations. Haploinsufficiency of the genes bone morphogenetic protein 4 (*BMP4*) and orthodenticle homeobox 2 (*OTX2*) accounts for most of the phenotypic abnormalities seen in these patients. There are only a few cases reported with Frias syndrome in the literature, and there are multiple variations present, which are not well recognized due to different set of genes involved. This case report presents the case of a young child with a deletion in 14q22.2q23.1 region containing both *BMP4* and *OTX2* genes as well as sineoculis homeobox homolog 1 (*SIX1*) and sineoculis homeobox homolog 6 (*SIX6*) genes. The case report illustrates the wide phenotypic findings associated with these genes along with additional unique findings that previously have not been commonly reported.

## Introduction

In a patient with 14q22q23 microdeletion syndrome, there is a wide range of genetic defects and associated morphological abnormalities. Pituitary anomalies may include pituitary hypoplasia/aplasia with growth hormone (GH) deficiency and growth retardation. Ocular anomalies may be anophthalmia/microphthalmia, exophthalmos, hypertelorism, ptosis, or absence of optic nerve/tracts. One may also see hand/foot anomalies, such as short digits, pes cavus, and polydactyly. Besides these three main characteristic anomalies, there are other clinical features that have been identified in these patients such as muscular hypotonia, intellectual disability, congenital genitourinary malformations, facial dysmorphism (microretrognathia, facial asymmetry, high-arched palate), and hearing loss. The clinical manifestations vary in each patient, and there is variable expression with smaller 14q22 deletions [1,2].

Some of the deleted genes include bone morphogenetic protein 4 (*BMP4*), orthodenticle homeobox 2 (*OTX2*), reticulon 1 (*RTN1*), sineoculis homeobox homolog 6 (*SIX6*), sineoculis homeobox homolog 1 (*SIX1*), and sineoculis homeobox homolog 6 (*SIX4*). *OTX2* plays an important role in the pituitary gland, brain, craniofacial, and sensory development. A major phenotype associated with *OTX2* mutations includes ocular defects like microphthalmia/anophthalmia and brain and pituitary malformations, as well as intellectual disability. *OTX2* gene defects are responsible for the second most common genetic cause of microphthalmia/anophthalmia, after *SOX2* gene mutations [1,2]. Bakrania et al. demonstrated *BMP4* gene expression in the formation of retina and lens, optic vesicle, digits, and pituitary gland, and thus mutations in this region can be responsible for ocular deformities like microphthalmia/anophthalmia, pituitary malformations, and poly/syndactyly [3]. Both *BMP4* and *SIX6* are known to likely contribute to the pituitary abnormality, while *SIX1* deletion is likely associated with abnormal craniofacial features [2]. Endothelin and *BMP4* signaling pathways responsible for facial features are regulated by *SIX1* and *SIX2* genes [4].

The case presented here includes a 7449 kb deletion in the 14q22.2q23.1 region containing several Online Mendelian Inheritance in Man (OMIM) genes including *BMP4*, *OTX2*, *SIX1*, and *SIX6*. The variable and distinctive presentation of the patient is highlighted and discussed in this case report.

## Case presentation

The case involves a female patient born in 2013 at the 39th week of gestation in a healthy family. The patient was found to have multiple complications, and the Apgar scores were 1, 3, and 5 at 1, 5, and 10 minutes, respectively. At birth, she had bilateral anophthalmia and persistent seizure activity. The patient required endotracheal intubation, positive pressure ventilation, chest compressions, and epinephrine, and the heart rate was detected at 7 minutes of age. She was then admitted to the neonatal intensive care unit where she was found to have jerky movements of the lower extremities and focal tonic-clonic movements of the left arm, as well as lip smacking. The patient was then given phenobarbital and was closely monitored and managed.

**Figure 1:**
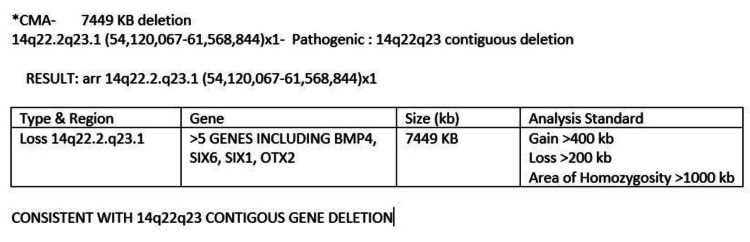
A detection of 7449 kb deletion was identified in chromosome 14q22.2q23.1. This region contains several OMIM genes including BMP4, OTX2, SIX1, and SIX6. OMIM, Online Mendelian Inheritance in Man; BMP4, bone morphogenetic protein 4; OTX2, orthodenticle homeobox 2; SIX1, sineoculis homeobox homolog 1; SIX6, sineoculis homeobox homolog 6.

At one month of age, genetic workup was performed, which revealed a 14q22q23 contiguous gene deletion (Figure 1).

The diagnosis of this patient was already made before the patient established medical care with the hospital. When the patient was three years old, she was brought to a pediatric cardiology clinic by her mother for a cardiology examination due to her history of a small secundum atrial septal defect and a restrictive ventricular septal defect. Her cardiac examination revealed a grade 3/6 harsh systolic ejection murmur, best heard at the lower left sternal border. The echocardiogram showed a small secundum atrial septal defect of 4 mm in diameter with left-to-right shunting and a small-to-moderate perimembranous ventricular septal defect measuring 3-4 mm in diameter with left-to-right shunting. There was no significant valvular dysfunction or structural abnormalities.

Patient has had multiple visits since then including emergency department visits for fever, infection, and respiratory failure. By age 7, her medical problems included congenital hydrocephalus s/p ventriculoperitoneal shunt, global developmental delay, hypopituitarism, anophthalmia, facial dysmorphism, seizure disorder, increased oropharyngeal secretions, failure to thrive in child, oropharyngeal dysphagia, severe protein-calorie malnutrition, tracheomalacia, status asthmaticus, and profound intellectual disability. She had no musculoskeletal deformities of the hand or foot. 

In the most recent visit, she underwent gastrostomy tube placement, where postoperatively she experienced several episodes of profound hypoxia and bradycardia related to her tracheomalacia, leading her to get a tracheostomy. The patient was critically ill due to the severity of the organ system dysfunction and was found to be infected with methicillin-resistant *Staphylococcus aureus* (MRSA) tracheitis. The patient passed away after a few days from sepsis at the age of seven.

## Discussion

**Figure 2:**
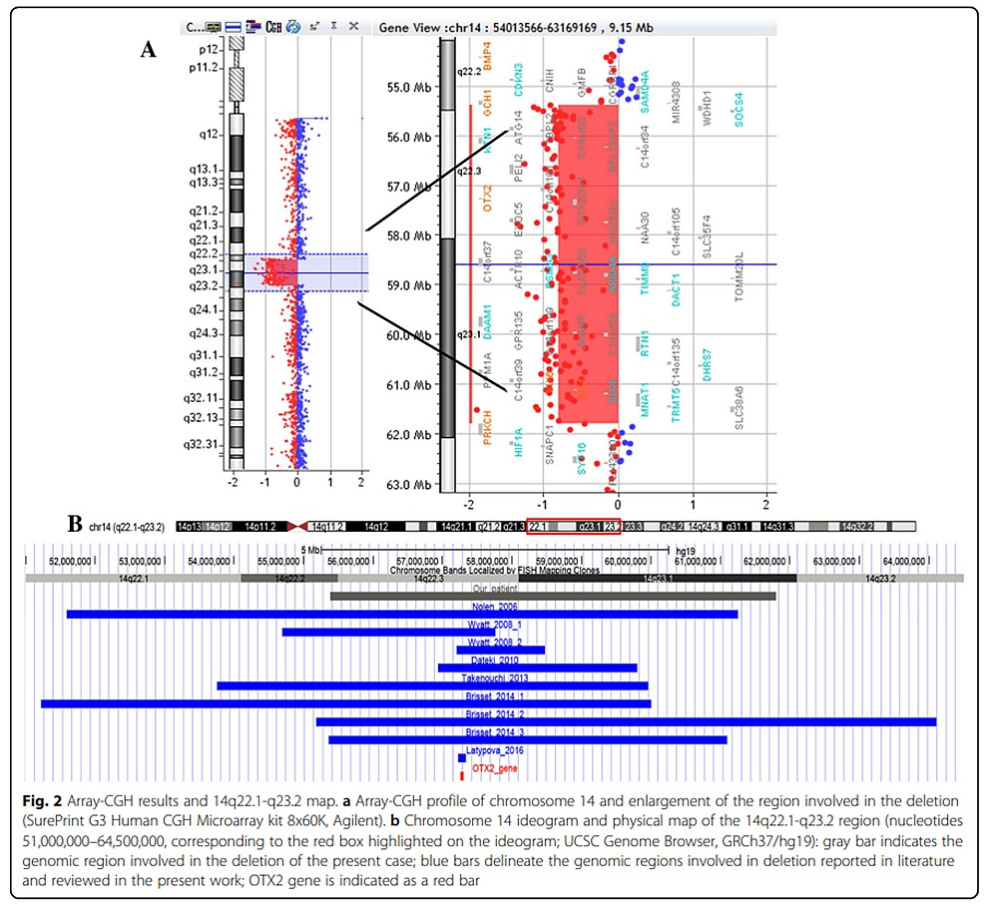
Chromosome 14 ideogram and physical map of the 14q22.1-q23.2 region. Image used under CC BY 4.0 license. Originally published by Pichiecchio, A et al. in BMC medical genomics journal. No changes were made [7].

Transcription factors including *SOX2*, *OTX2*, and *BMP4* are responsible for morphological development of the brain, ocular structures, optic nerve, and the optic tracts. Specifically, *SOX2* mutations are associated with anophthalmia-esophageal-genital syndrome, and *OTX2* mutations cause anophthalmia and pituitary abnormalities. Anophthalmia, pituitary abnormalities, and polysyndactyly are also associated with *BMP4* mutations [1]. Congenital anophthalmia is generally followed by pituitary abnormalities and hormonal imbalance including GH deficiency leading to growth failure [2]. In patients with *OTX2* mutations, the coexistence of ocular manifestations and GH deficiency is the highest (30%) [5]. Additionally, *BMP4* mutations also lead to a similar ocular and brain phenotype [6]. Pichiecchio et al. provide an analysis of the chromosome 14 ideogram and a physical map of the 14q22.1-q23.2 genes involved in this disease process (Figure 2) [7].

Both *OTX2* and *BMP4* are located in the region of 14q22q23, and mutations including deletions and single-nucleotide substitutions have been reported in these genes. Not all phenotypic features are well characterized in these rare genetic mutations, and patients may have different presentations and symptoms. A patient with Frias syndrome due to microdeletions in 14q22.2q23.1 region is identified in this case report to help better understand the wide and variable phenotypic spectrum of 14q22q23 deletions.

These mutations are responsible for a broad spectrum of structural and functional abnormalities with highly variable phenotype, proportional to the numerous cell differentiation and migration pathways involved in these genes. Following is the description of the patient’s range of clinical manifestations and presentations relative to the body systems.

**Figure 3:**
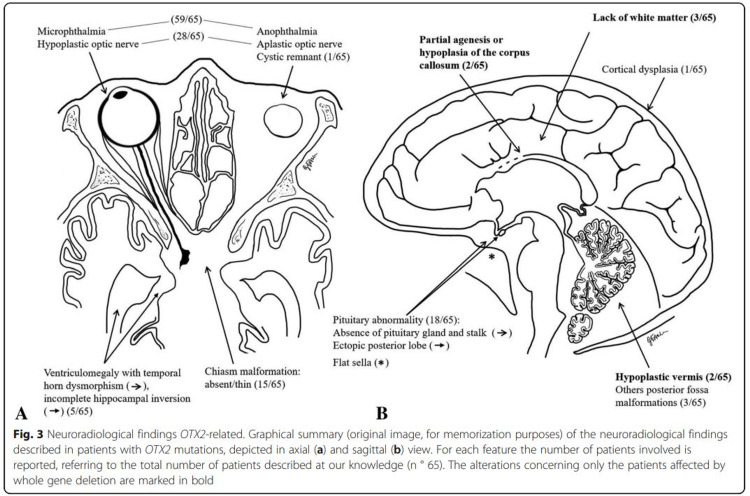
Neuroradiological findings associated with OTX2 mutation. Image used under CC BY 4.0 license. Originally published by Pichiecchio, A et al. in BMC medical genomics journal. No changes were made [7]. OTX2, orthodenticle homeobox 2.

**Figure 4:**
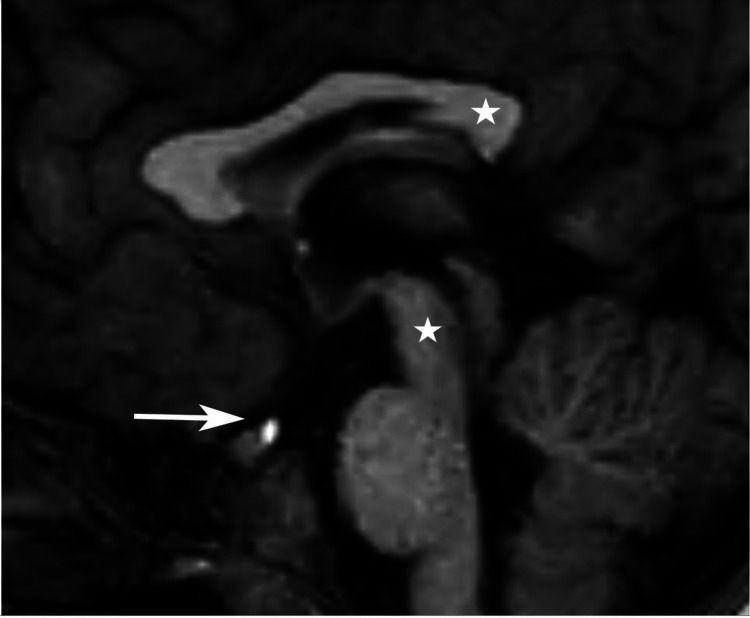
Sagittal T1 weighted image demonstrating high riding position of posterior pituitary, above the level of sella turcica (white arrow). Also note the mid brain parenchymal volume loss and foreshortening of the splenium (white stars).

**Figure 5:**
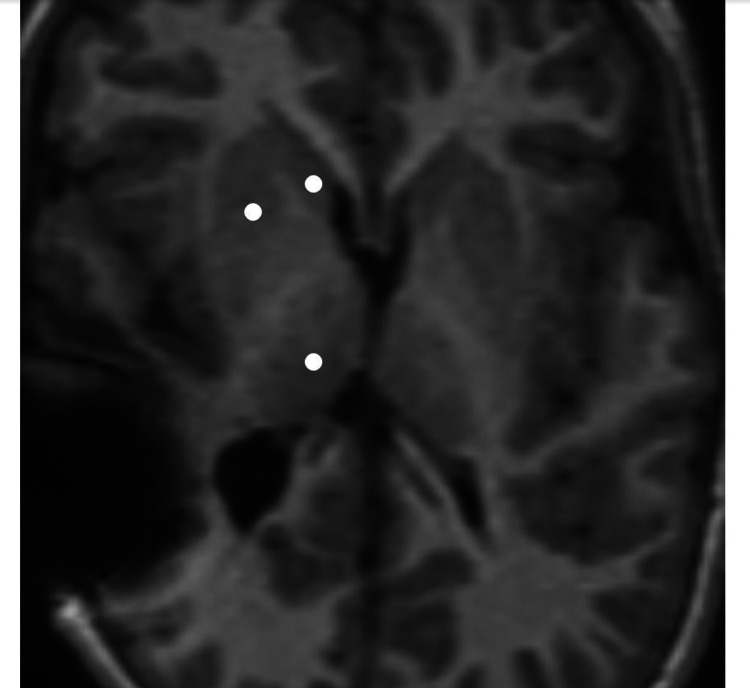
Axial T1 weighted images demonstrate relative paucity of white matter in a seven-year-old patient, specifically in the periventricular location. In addition, there is some remarkable volume loss of deep gray matter structures bilaterally including head of caudate, lentiform nuclei, and thalami (white discs).

The neurological manifestations of the case presented included congenital hydrocephalus, seizure disorder, global developmental delay, and hypopituitarism. *OTX2* and *BMP4* are two significant genes responsible for the structural development of brain, and mutations result in a variable morphological abnormality, especially associated with the pituitary gland (Figure 3). GH is the most vulnerable hormone and is most commonly affected due to mutations in these genes. It has been shown that even with normal-size pituitary gland, GH can be deficient since *OTX2* also helps regulating the secretion of gonadotrophin-releasing hormone by the hypothalamus [2,7]. Pituitary dysfunction can either include aplastic or hypoplastic anterior and/or posterior lobes or interrupted or absent pituitary stalk [2]. *OTX2* mutation can also lead to reduced/atrophic white matter, dilated ventricles, abnormal hippocampal gyration, malformations of the posterior cranial fossa, and/or corpus callosum agenesis [8,9]. This case’s radiographic images revealed a high riding position of posterior pituitary and remarkable volume loss of deep gray matter structures (Figures 4, 5). She was found to have global developmental delay and intellectual disability secondary to the genetic disorder or partly due to hypoxia at birth (cardiopulmonary resuscitation was required up to 7 minutes), as well as a seizure disorder and hydrocephalus.

**Figure 6:**
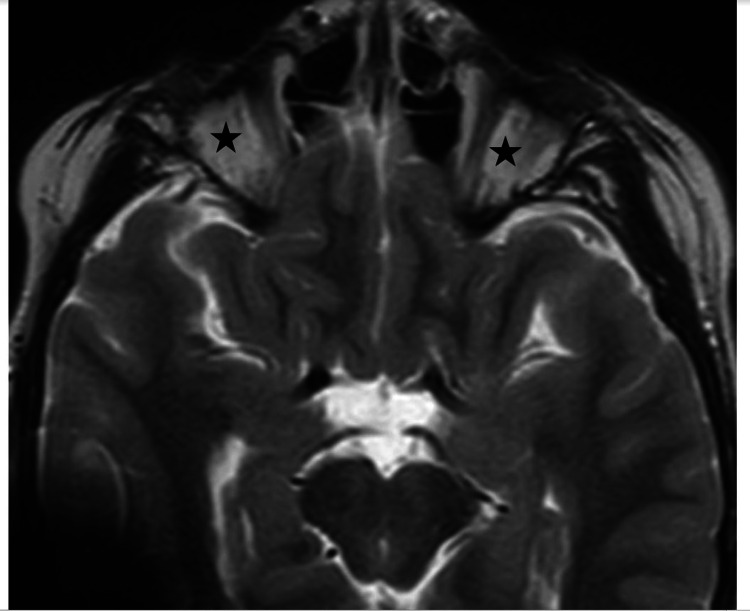
Axial T2 weighted images confirm absence of globes bilaterally (black stars), compatible with diagnosis of anophthalmia.

Similarly, there are numerous facial and ophthalmologic manifestations mainly associated with genes *SIX1*, *SIX2*, *OTX2*, and *BMP4*. Eye and optic nerve development are affected by *OTX2* mutations [10]. Other implications including visual pathway dysgenesis/agenesis such as the chiasmatic hypoplasia/aplasia have been found [7]. *SIX1* and *SIX2* encode for transcription factors and, if disrupted, can lead to craniofacial abnormalities [4]. Patient in this case presented with bilateral anophthalmia (Figure 6) at birth and rudimentary optic nerves, as well as facial dysmorphism.

In addition, there are other significant manifestations found in this case that have previously not been commonly associated with 14q22q23 microdeletion syndrome. These include the alimentary tract, respiratory, and cardiac findings. Increased oropharyngeal secretions, oropharyngeal dysphagia, severe protein-calorie malnutrition, tracheomalacia, and status asthmaticus were some of the conditions diagnosed in this patient. The patient was transitioned to feeding tubes multiple times and underwent a tracheostomy, and, eventually, developed MRSA tracheitis. Although moderate, atrial septal defect and ventral septal defect were among the cardiac findings in this patient. Furthermore, polydactyly/syndactyly (musculoskeletal deformities), which is one of the hallmarks of 14q22q23 microdeletion syndrome, was never clinically observed or documented in this patient’s reports. Thus, there are noteworthy differences observed in this case compared to other cases reported in the literature, adding to the already broad phenotypic spectrum of findings in this syndrome.

## Conclusions

This case report describes the wide spectrum of phenotypic findings in a patient with 14q22q23 microdeletion syndrome, also called Frias syndrome. There have been only a few cases reported in the literature describing the clinical presentation and manifestations of these patients. This case report adds to the phenotypic spectrum by covering the gastrointestinal, respiratory, and cardiac findings in addition to the neurologic, ophthalmic, and endocrine findings. Being aware of the wide range of manifestations in these patients is important for appropriate diagnostic tests to properly diagnose and manage the multiple associated disorders.
